# Lactogenic differentiation of HC11 cells is not accompanied by downregulation of AP-2 transcription factor genes

**DOI:** 10.1186/1756-0500-4-345

**Published:** 2011-09-09

**Authors:** Richard Jäger, Leontios Pappas, Hubert Schorle

**Affiliations:** 1 Apoptosis Research Centre, School of Natural Sciences, National University of Ireland, Galway, Ireland

## 

Following the publication of our article [1], an error in Table 1 was noted. The primer sequences used for PCR of ß-casein are incorrectly stated.

The ß-casein primer pair has been incorrectly stated as:

ß-casein sense: 5'-CCATCCTGCGTCTGGACCTG-3'

ß-casein antisense: 5'-GGAATGTTGTGGAGTGGCAG-3'

The correct ß-casein primer pair that has been used in the study is:

ß-casein sense: 5'-GCCTTGCCAGTCTTGCTAAT-3'

ß-casein antisense: 5'-GGAATGTTGTGGAGTGGCAG-3'

We apologise for any inconvenience this may have caused.

## References

[B1] Jäger R Pappas L Schorle H Lactogenic differentiation of HC11 cells is not accompanied by downregulation of AP-2 transcription factor genes BMC Res Notes 2008 23;1 29 18710545 10.1186/1756-0500-1-29 PMC2518283

